# Molecular answers to the high failure rate of malaria RDTs

**DOI:** 10.1186/1475-2875-9-S2-P56

**Published:** 2010-10-20

**Authors:** John Waitumbi, Nancy Nyakoe, Linda Muringo, Ismail Mahat, Moses Otiende

**Affiliations:** 1 Kenya Medical Research Institute/Walter Reed Project, Kisumu, Kenya

## Background

Rapid diagnostic tests (RDTs) for malaria are increasingly being used for management of patients. Different studies have shown significant failure rate of RDTs, especially in children and in areas of low malaria transmission. In this study, we sort molecular answers to RDT failure by re-screening RDT negative samples for malaria at genus and species level as well as for diversity of *Plasmodium falciparum* histidine-rich protein 2 (PfHRP-2) and lactate dehydrogenase (pLDH). PfHRP-2 and pLDH are the malaria target antigens for the commonly used commercial RDTs.

## Methods

Patients (n=1054) with high fever but negative for malaria by RDT were recruited for the study from a network of clinical sites in Kenya. For RT-qPCR malaria diagnosis, total nucleic acids were isolated from whole blood and evaluated by a strategy that combines RNA and DNA using 18 S rRNA primers and probe for malaria genus and species. For diversity of PfHRP-2 and pLDH genes, PCR were subjected to gel electrophoresis to determine amplicons sizes.

## Results

Of the 1054 RDT negative febrile patients, malaria at genus level was detected in 160 patients (15%). Of the 160, half (52%) had a parasite density below the limit of detection of RDT in use at the hospital (100 parasites/ μL). On speciation, 60% of the patients had *P. falciparum,* either alone or in combination with *P. malarie (7%)*, or *P. ovale* (2%). 31% of genus positive malaria could not be speciated. Of the *P. falciparum,* 56% had full size PfHRP2 gene (927 bp), 40% had truncation of up to 325 bp and 1 % appeared not to have PfHRP2 gene at all. All parasites had full size pLDH (922 bp) gene.

## Conclusion

It is concluded that, 50% of the RDT failures are attributable to the detection threshold of the RDTs. It is unclear what impact the truncation of PfHRP2 gene has on sensitivity of RDT. That the failure to detect *P.ovale* and *P.malarie* is due to low parasite density commonly associated with these infections and not due to truncation of pLDH gene. Lastly, the unspeciated malaria infections are probably due to higher sensitivity of genus compared to species RT-qPCR.

